# Immobilization of Hexavalent Chromium Using Self-Compacting Soil Technology

**DOI:** 10.3390/ma15062335

**Published:** 2022-03-21

**Authors:** Zymantas Rudzionis, Arunas Aleksandras Navickas, Gediminas Stelmokaitis, Remigijus Ivanauskas

**Affiliations:** 1 Faculty of Civil Engineering and Architecture, Kaunas University of Technology, Studentų Str. 48, 51367 Kaunas, Lithuania; arunas.navickas@ktu.lt (A.A.N.); gediminas.stelmokaitis@ktu.lt (G.S.); 2 Faculty of Chemical Technology, Kaunas University of Technology, Radvilėnų pl. 19, 50254 Kaunas, Lithuania; remigijus.ivanauskas@ktu.lt

**Keywords:** immobilization, hexavalent chromium, self-compacting soil

## Abstract

A study of immobilization of hexavalent chromium in the form of Na_2_CrO_4_ salt by self-compacting soils (SCS) is presented. Carbofill E additive was used as SCS binder. The efficiency of immobilization of Cr (VI) was evaluated by washing out chromium compounds from SCS samples. The influence of the nature of the soil and the content of Carbofill E and Na_2_CrO_4_ in the SCS samples on the efficiency of Cr (VI) immobilization was studied. It was found that the nature of the soil and the content of Carbofill E in the SCS samples affect the immobilization of Cr (VI). Moreover, increasing the Carbofill E content in SCS samples further increases Cr (VI) immobilization. X-ray diffraction studies of the samples with immobilized hexavalent chromium showed that part of the sample transforms from a readily soluble form of salt into oxide forms of chromium and calcium-chromium, which are practically insoluble in water.

## 1. Introduction

Self-compacting soil mixture (SCS) is a highly flowable soil mixture prepared by homogeneous mixing of excavated loose soil, binder, and various additives. The mixture compacts its own weight and easily fills various excavations after formwork removal, cavities, and other hard-to-reach areas without the use of vibration. The technology for the production of SCS is in accordance with the principles of sustainable and environmentally friendly technology. By using SCS mixtures in the construction of buildings and roads, we conserve the natural resources of gravel/sand mixtures and it is possible to use excavated soil in the construction site for the same needs as we ordinarily use sand and gravel. Therefore, it is possible to recycle hardened SCS and make a product from it with new properties needed to implement design solutions. Heavy metals can be immobilized in SCS to permissible regulatory limits, thus providing a solution for contaminated soil remediation problems.

Industrialization and technological progress increase the release of hazardous waste, such as heavy metals, metalloids, and organic pollutants, into the environment [1,2]. Heavy metals are known to be significant environmental contaminants due to their high density and toxicity at low concentration. Furthermore, they cannot be decomposed into a non-toxic form. Most of the heavy metal concentration accumulates in the sediments of water basins and at the upper level of the soil. The main pathway of exposure to heavy metals for humans is food chain contamination [3,4]. Long-term contact with heavy metals causes major health problems in humans [5,6], animals [7], and plants [8]. Due to this, public health and environmental concerns are constantly raised. Therefore, it is imperative to reduce heavy metal pollution and the possibility of food contamination. Due to the widespread use of chromium in industrial processes, it is a major pollutant, among other toxic heavy metals. It is used mainly in electroplating [9,10], plating [11], and the manufacture of steel [12], tannery [13,14], and textiles [15]. Therefore, for these reasons there is a possibility that large amounts of chromium may enter the environment. Moreover, chromium is found naturally in small amounts together with other metals, like iron. Chromium has a wide range of oxidation states (−4 − +6). The most stable and common forms are trivalent chromium (Cr(III)) and hexavalent chromium (Cr(VI)). They are chemically stable and found naturally in the environment [16]. Cr(VI) and Cr(III) differ in their oxidation states as well as in their toxicity. Cr(III) has been shown to be an essential microelement in the diet of living organisms and is important for the metabolism of lipids and glucose [17,18]. Cr(VI) is known to be a toxin in nature, and small amounts can easily enter cell membranes [19]. Both acute and chronic exposure to Cr(VI) can cause numerous diseases such as pneumonia, bronchitis, gastrointestinal hepatic disorders, hypersensitivity, and renal impairments [16]. Human studies have shown that Cr(VI) is a carcinogen and increases the risk of lung cancer when inhaled [20].

Therefore, public health studies and environmental issues have increased interest in the removal of traces of chromium compounds from soil, surface, and groundwater over the past several decades. During the last decade, many physical, chemical, and biological treatment methods have been used to remove chromium compounds from contaminated soils, sediments, and water. Conventional methods, such as chemical precipitation [21,22], adsorption [23,24], coagulation [25], ion exchange [26,27], and membrane separation [28,29] have been used to remove chromium-containing contaminants from industrial waste. Unfortunately, most of the methods listed above are only effective in removing chromium compounds from liquid media. The detoxification method for chromium-contaminated soils and sediments is based on chromium-containing compounds that transform to a form with solubility below the threshold for nonhazardous waste. Geopolymer metakaolin [30], Portland cement [31], hydroxyapatite [32], gypsum [33], alkali-activated fly ash [34], spinel-based glass-ceramic [35], and carbon monoxide [36] are widely used for this purpose. Despite the success of using these reagents for the detoxication of chromium-containing contaminants, they do face certain disadvantages, such as high cost, low efficiency, large amounts of recovered sludge, the resulting severe limit of their transportation and storage, etc. However, this can be overcome by upgrading the detoxication of chromium-containing contaminants using SCS technology. The most toxic hexavalent chromium and its content can be disposed of using a Carbofill E additive as a binder component.

Other components of the soil also have an influence on its final properties. Soils with a ‘cement-stabilized clay admixture can significantly improve water stability’ [37]. Gravelly sand stabilized with cement [38] can achieve optimal compaction parameters and meet the requirements as a material for the subbase of the road. Boundary conditions for self-compacting clay concrete and the possibilities to achieve identical results with a material poured into formwork rather than compacted were presented [39].

As heavy metals are very hazardous substances with long-term effects on ecosystems, their leaching from SCS is important not only from an ecological point of view, but also from the perspective of the use of these soils. Leaching of heavy metals from SCS within the permitted emission limits would allow its widespread use not only where it is normally used, but also in the management of contaminated sites, where the ‘bound’ pollutants do not enter the groundwater.

## 2. Materials and Methods

### 2.1. Materials and Characterization of Raw Soil (Materials)

All chemicals used were of reactive or analytical grade. Potassium dichromate (K_2_CrO_4_) (>99.0%, from Merck ACS, St. Louis, MO, USA) was used. The water for all experiments was obtained from the Water Stills GFL 2008 (Burgwedel, Germany) system.

The binder material Carbofill E (Thomas Cement, Germany) was used for experiments, which is a factory-produced mineral, single-component binder system that is used, usually without adding any other components, to produce liquid soils/temporarily flowing, self-compacting fillers based on excavated soil, tested recycled material or aggregates. The main Carbofill E binder compounds consisted of hatrurite (Ca_3_ (SiO_4_)) and α-quartz SiO_2_, tricalcium aluminate (Ca_3_Al_2_O_6_), gismondine (CaAl_2_Si_2_O_8_·4 (H_2_O)), calcium sulfate (CaSO_4_). The initial setting time (Vicat) of the Carbofill E mixture was 270 min., and the final setting time was 370 min. 43.5% water amount from cement mass was needed to prepare the paste of standard consistency. The flexural strength of the cement mortar with Carbofill E was 2.09 MPa after 3 days and 2.2 MPa after 28 days. The compressive strength of this cement mortar with Carbofill E was 26.5 MPa after 3 days and 46.2 MPa after 28 days.

For this study, clay and sandy soil were used from local construction sites in Lithuania. The sandy soil particles were sieved (according to EN 932-1), and their particle size distribution is given in Table 1. 

According to the results of granulometric composition tests of sandy soil, it is classified as poorly coarse sand according to EN ISO 14688-1.

The Atterberg limits (plastic limit, PL and liquid limit, LL) were found to determine the clay soil plasticity index according to EN ISO 17892-12. Variations of the Atterberg limits (the cone penetrometer method was used) of the clay are given in Table 2. The plasticity index (PI) is a measure of the plasticity of clay and according to the value of PI (25.36%) this clay could be assigned to highly plastic soils. The plasticity index (PI) is the difference between the liquid limit and the plastic limit (PI = LL−PL). The PI (>17) indicates highly plastic soil (based on EN ISO 14688-2).

The water permeability of clay and sandy soils was determined according to EN ISO 17892-11. Permeability of raw soils: sandy soil—5 × 10^−5^ m/s, and clay soil—5 × 10^−10^ m/s. 

Soil pH was measured in a soil-water slurry. The procedure was performed according to ISO 10390 using a pH meter (Hanna instruments HI 98130). The average measurement results are sand pH-8.16, clay pH-7.98.

Organic matter content was determined after drying and combusting (450 °C) the samples according to EN 13039. The content of organic matter found in sand was 0.32% and 0.51% was found in clay. Therefore, we can say that there is no organic matter in the soil.

### 2.2. Methods

The mixing process was kept constant in the production of SCS to achieve the same homogeneity and uniformity in all the mixtures. It started by mixing all the soil and Carbofill E for one minute using a standard mixer as described in EN 12390-4. Then, three-quarters of the mixing water with Cr (VI) was added and mixed additionally for one minute. Thereafter, the required amount of water was added and the SCS was mixed additionally for approximately three minutes. After the mixing process was completed, the test was carried out on fresh SCS mortar to assess the diameter of the slump flow and the density of the mixture. The diameter of the slump flow was measured according to the procedure recommended by EN 12350-8. The diameter of the SCS mortar is measured in two perpendicular dimensions and the average is reported as the final diameter. The SCS mortars were proportioned to give a slump flow of 600 to 650 mm, which was achieved by using different water content. Between 5 × 10^2^ and 1 × 10^5^ mg Cr (VI) per kg of the mixture was added to the SCS samples during their formation. Na_2_CrO_4_ salt dissolved in water was used as a source of Cr (VI). It was found that dissolved Cr (VI) had no effect on the physical—mechanical properties of the SCS.

The hardened SCS samples were also tested for compressive strength. Measurements were made after 7, 14, 28, 56, and 91 days, following the EN 12390-4 standard. Three 100 mm cubes were used for compressive strength tests. The cube samples were cast into the moulds without any compaction and vibration. After demoulding, all samples were kept at 20 ± 2 °C and 95% humidity until testing.

The permeability of hardened SCS samples was measured according to the procedure recommended by EN ISO 17892-11. Measurements were made after 28 days. The SCS samples were prepared as cylindrical samples (ø = 71,4 mm, H = 35 mm) without any compaction and vibration. All samples were stored at 20 ± 2 °C and 95% humidity until testing. The falling head test is performed when the test primers are poorly water-permeable (according to EN ISO 17892-11).

**Figure 1:**
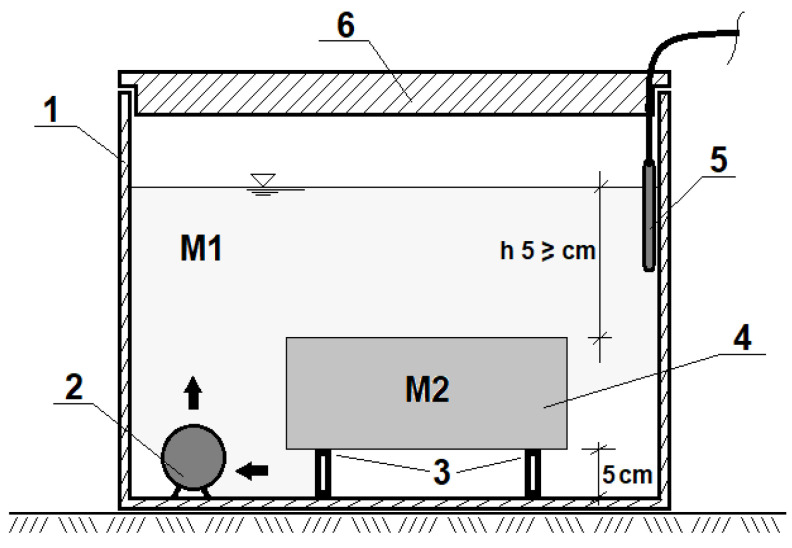
Scheme for washing out Cr (VI) compounds from self-compacting soils (SCS) samples: 1—polyethylene container; 2—circulating water pump to create a flow of flushing water; 3—SCS supporting structure; 4—sample SCS; 5—thermostat for maintaining constant temperature; 6—cover; M1—sample wash water, M2—sample.

The washing out of Cr (VI) compounds from SCS samples was carried out in accordance with the recommendations of the standard EN 12457-2-2003. Two methods were used to bring the washing of Cr (VI) compounds as close as possible to real environment conditions. Method N 1 simulates the washout of undisturbed compacted soil. Here, the SCS cubes were continuously rinsed for 24 h in 10 L of distilled water circulated at 20 °C (Figure 1). The water was then filtered through a 0.45 µm filter and the Cr (VI) content was determined. Method N 2 simulates the washing out of crushed soil. For this, the SCS cube was crushed and a grain fraction ≤4 mm was taken for further analysis. They were dried at 105 °C to constant weight and cooled to 20 °C; 50 g of these grains were placed in a conical flask filled with 500 mL of distilled water and shaken on a shaker at 200 rpm and 20 °C for 24 hours. The water used to wash the grains was analyzed as in the first method.

The total amount of chromium in the water washed out from the SCS samples was determined by using the atomic absorption spectrometer (AAS) AA–7000 Shimadzu (Japan) (featuring λ= 357.9 nm) equipped with an electrodeless discharge lamp and an air-acetylene flame. For the conditions described above, the sensitivity of the AAS method is ~0.2 µg/mL chromium for 1% absorption [40].

## 3. Results and Discussion

### 3.1. Physical and Mechanical Research of the SCS

The test results relevant to the diameter of the slump flow and the density of the mixtures are presented in Table 3. All SCS mixtures were designed to reach a diameter of the slump flow of 600–650 mm, which was acquired by adjusting the dose of water used.

**Figure 2:**
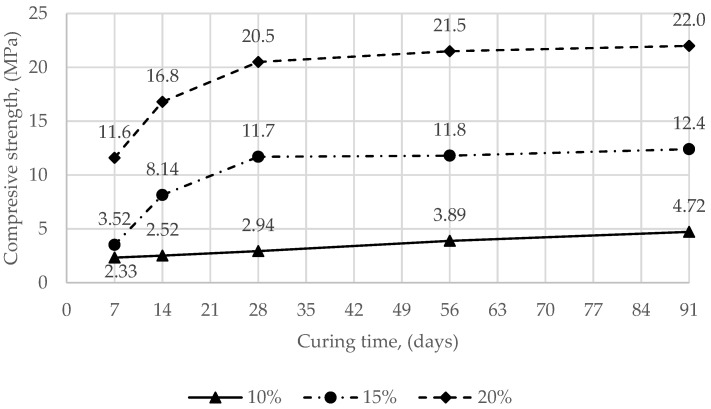
Compressive strength (samples of SCS with sandy soil and 10%, 15%, 20% Carbofill E binder) development with time.

**Figure 3:**
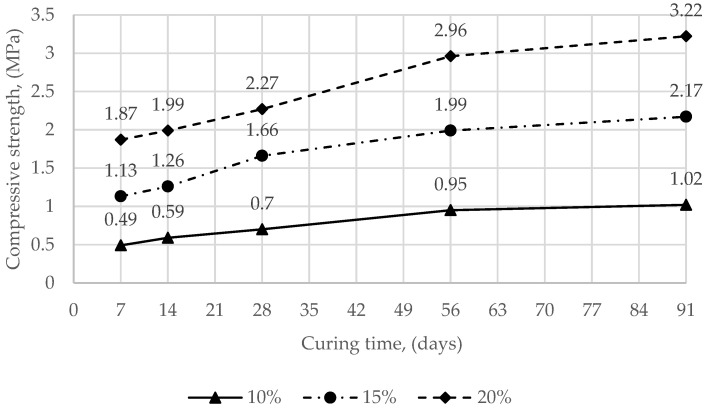
Compressive strength (samples of SCS with clay soil and 10%, 15%, 20% Carbofill E binder) development with time.

The compressive strength test results of different mixture compositions are shown in Figure 2 and Figure 3. The average compressive strength of the SCS mixed with sandy soil and 10%, 15%, 20% Carbofill E ranged from 2.33 to 22.0 MPa (Figure 2). The average compressive strength of the SCS mixed with clay soil and 10 %, 15%, 20% Carbofill E ranged from 0.49 to 3.22 MPa. (Figure 3).

**Figure 4:**
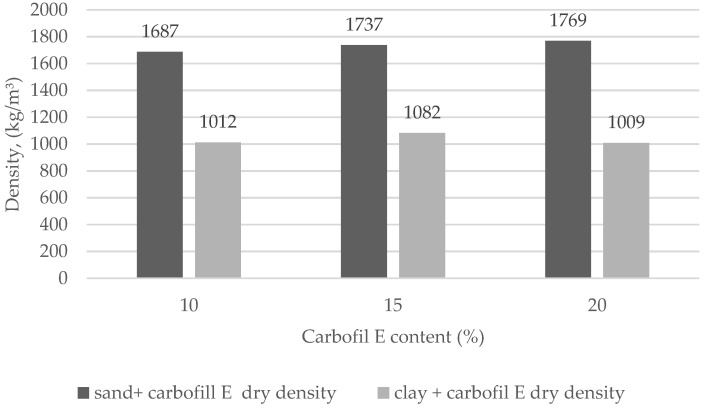
Density of SCS with sandy and clay soils corresponding to the amount of Carbofill E binder.

Figure 4 illustrates the average dry density of SCS mixed with sandy soil and 10%, 15%, 20% Carbofill E, and with clay soil and 10%, 15%, 20% Carbofill E of the samples obtained from each mixture after 28 days of hardening. The average dry density of sandy soil and Carbofill E samples is in the range of 1687 kg/m^3^ to 1769 kg/m^3^, the density of clay soil and Carbofill E samples is in the range of 1009 kg/m^3^ to 1082 kg/m^3^. The lowest density was reached in the sample made of clay and Carbofill E mixtures.

**Figure 5:**
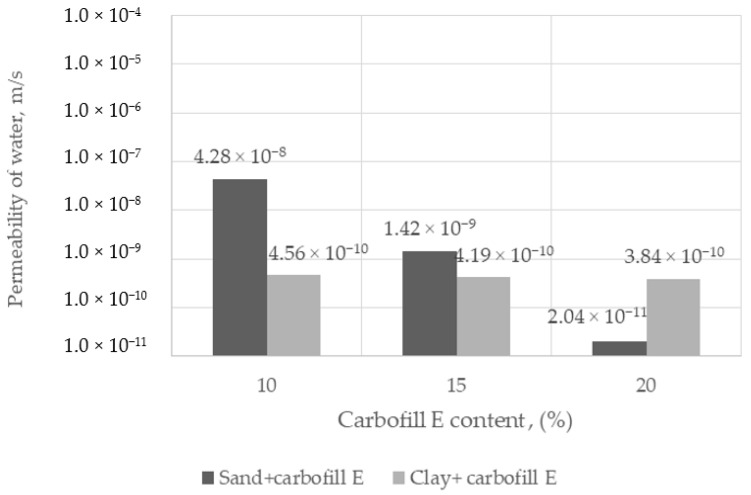
Water permeability of SCS with sandy and clay soil corresponding to the amount of Carbofill E binder.

The water permeability of hardened SCS with sandy and clay soils is shown in Figure 5. The coefficient of permeability of SCS mixed with sand and 10 %, 15%, 20% Carbofill E was found to vary between 4.28 × 10^−8^ m/s and 2.04 × 10^−11^ m/s, while the coefficient of permeability of SCS mixed with clay and 10 %, 15%, 20% Carbofill E was found to vary between 4.56 × 10^−10^ m/s and 3.84 × 10^−10^ m/s.

### 3.2. Immobilization Studies of SCS Contaminated Cr (VI)

**Figure 6:**
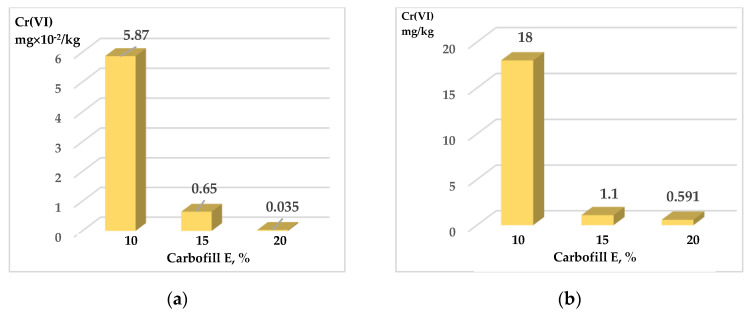
Washing out of chromium compounds from SCS sandy samples with immobilized Cr (VI) 3000 mg/kg and various amounts of Carbofill E. Samples: uncrushed—(**a**); crushed—(**b**).

**Figure 7:**
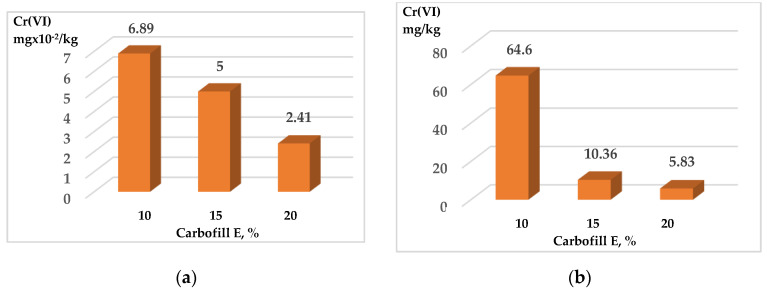
Washing out of chromium compounds from SCS clay samples with immobilized Cr (VI) 3000 mg/kg and various amounts of Carbofill E. Samples: uncrushed—(**a**); crushed—(**b**).

The research was carried out on the sandy and clay soils most widespread in Lithuania. Since the excavated sites in landscaped plots can be improved with SCS in the future, it is important that heavy metals are securely immobilized and their leeching from these soils is minimized. Initially, the effect of Carbofill E additive on the washing out of chromium compounds from SCS samples was evaluated. The Cr (VI) content in the samples exceeded the allowed hygienic standards (3000 mg/kg) by 30 times [41], and the content of Carbofill E additive ranged from 10% to 20%. Compared with other studies [42], the same amount of the additions in blended cement pastes, which contain 15–20% bagasse ash, can be used effectively for the immobilization of concentrated waste Cr(VI) solutions. The data presented in Figure 6 and Figure 7 show that the addition of Carbofill E dramatically reduces the washing out of chromium from all modified soil samples. For example, only 18.0 and 64.6 mg/kg of chromium were washed out from crushed sandy and clay SCS samples with 10% Carbofill E, respectively (Figure 6b and Figure 7b). Meanwhile, the amount of chromium compounds washed out from uncrushed SCS soil samples decreased hundreds of times to 5.87 × 10^−2^ and 6.89 × 10^−2^ mg/kg, respectively (Figure 6a and Figure 7a). A further increase in the Carbofill E content in the SCS samples further reduced the chromium washing out, although not as noticeably. For example, an increase in the concentration of Carbofill E in uncrushed sandy SCS samples from 10% to 15 % reduced the washing of chromium by approximately 9 times (from 5.87 × 10^−2^ to 0.65 × 10^−2^ mg/kg) and more than 16 times (from 18.0 to 1.1 mg/kg) in crushed samples. An increase in the amount of Carbofill E up to 20% in sandy SCS reduced the washing out of chromium from both soil samples only 1.86 times: from 0.65 × 10^−2^ to 0.35 × 10^−2^ mg/kg in uncrushed samples and 1.1 up to 0.591 mg/kg in crushed samples, respectively. The same dependence, only with a relatively large washing out of chromium, was observed for clay SCS samples (Figure 7). The higher efficiency of chromium immobilization in sandy soil compared to clay soil is due to its higher density (Figure 4). As a result, the water permeability of the soil de-creases, which leads to a decrease in the leaching of chromium from sandy soil (Figure 5).

**Figure 8:**
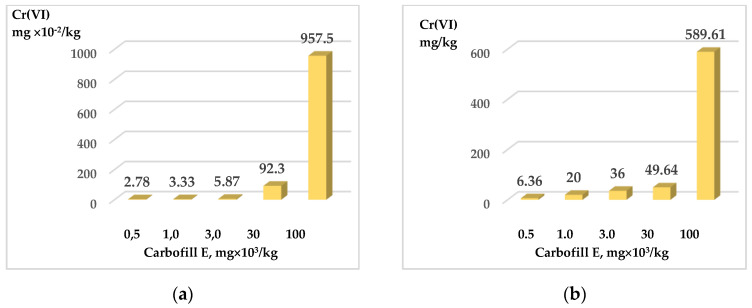
Washing out of chromium compounds from SCS sandy samples with immobilized various amounts of Cr (VI) and 10% Carbofill E. Samples: uncrushed—(**a**); crushed—(**b**).

Since 10% Carbofill E in the sandy and clay SCS samples is sufficient to immobilize Cr (VI), further studies were performed on samples with 10% of this binder. The sandy samples of SCS with immobilized Cr (VI) content exceeding the permissible level from 5 to 1000 times have been investigated. As can be seen from the graphs in Figure 8, the maximum chromium content (589.61 mg/kg) was washed out only from the crushed sample, which had a maximum chromium content (10^5^ mg/kg) (Figure 8b). Meanwhile, even at such high levels of chromium, only 9.58 mg/kg was washed out from the undisturbed SCS sample.

The effectiveness of immobilization of heavy metals in SCS soils can be better illustrated not by the absolute amount of washed-out metals, but by the percentage of heavy metals remaining in these soils after the washing out procedure of the total amount added to them. Data in Table 4 show that the nature of the soil affects the immobilization of Cr (VI) only in crushed SCS samples with a 10% addition of Carbofill E. Meanwhile, in uncrushed soil samples, regardless of the nature of the soil and the amount of Carbofill E, more than 99.99% of the total added Cr (VI) content (3000 mg/kg) was immobilized. In sandy SCS samples with 10% Carbofill E and the maximum Cr (VI) content (10^5^ mg/kg), the immobilization efficiency did not change: it was more than 99.99% in the uncrushed sample and 99.4% in the crushed sample.

Studies showed that Carbofill E supplementation has an inhibitory effect on the immobilization of Cr (VI) in SCS. Meanwhile, the nature of the soil insignificantly affects the immobilization of Cr (VI) only in the samples of crushed soil.

### 3.3. X-ray Diffraction Studies of SCS Contaminated Cr (VI)

**Figure 9:**
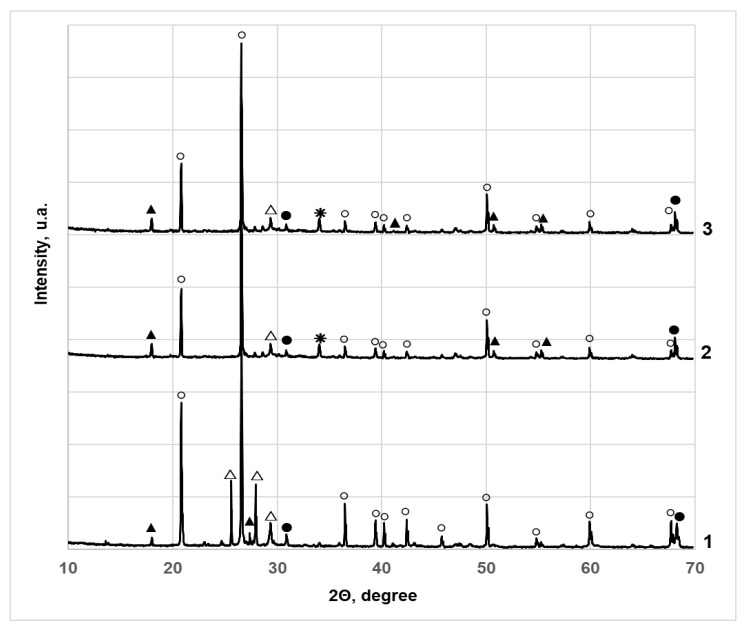
X-ray diffractograms of sandy SCS samples with different amounts of additives: 1–10% Carbofill E and Cr (VI) 3000 mg/kg; 2–20% Carbofill E and Cr (VI) 3000 mg/kg; 3–10% Carbofill E and Cr(VI) 30,000 mg/kg. The peaks were identified and assigned as follows: (○) quartz SiO_2_ 83–539; (▲) wollastonite 2M (*monoclinic*) CaSiO_3_ 84–655; (△) wollastonite 2M (*monoclinic*) Ca_3_(Si_3_O_9_) 76–1846; (●) calcium chromium oxide (*hexagonal*) Ca_5_(CrO_4_)_3_O_0.5_ 38–294; (**✳**) chromium oxide (*rhombohedral*) Cr_2_O_3_ 84-315.

**Figure 10:**
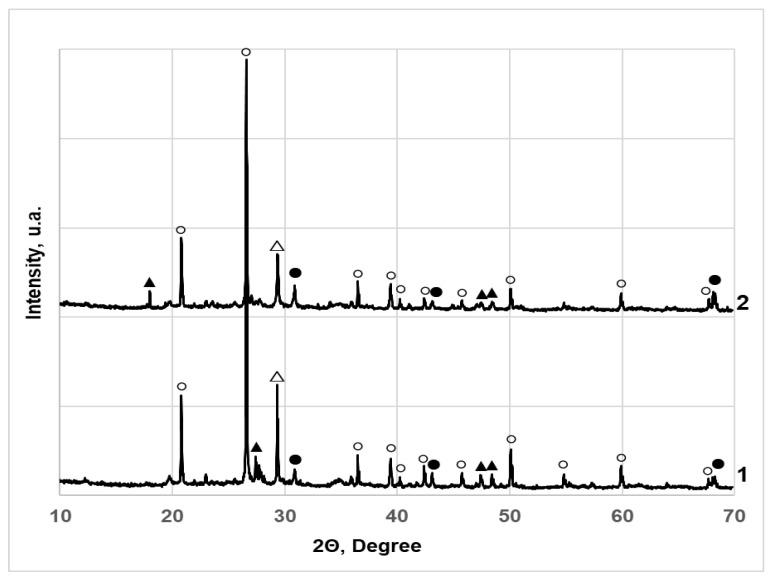
X-ray diffractograms of clay SCS samples with Cr (VI) 3000 mg/kg and different amounts of Carbofill E: 1–10% 2–20%. The peaks were identified and assigned as follows: (○) quartz (*hexagonal*) SiO_2_ 83–539; (▲) wollastonite 2M (*monoclinic*) CaSiO_3_ 84–655; (△) wollastonite 2M (*monoclinic*) Ca_3_(Si_3_O_9_) 76–1846; (●) calcium chromium oxide (*hexagonal*) Ca_5_(CrO_4_)_3_O_0.5_ 38–294.

To understand the nature of the immobilization of Cr (VI) compounds in SCS, XRD analysis of these soils was carried out. This is a nondestructive method of instrumental analysis, which is informative and suitable for characterizing the crystal structure of materials; therefore, it is widely used in the study of inorganic crystalline compounds (silicates and other minerals, ceramics, cement and its hydration products, zeolites). The results of the study are presented in Figure 9 and Figure 10, and the peaks of the identified compounds and their positions are tabulated in Supplementary Material Tables S1–S5. As expected, typical diffraction peaks for hexagonal quartz SiO_2_ (83-539) prevail in all investigated samples of sandy SCS (Figure 9). Furthermore, their number and intensity decrease with increasing concentration of the additive (Tables S1–S3). Also, two peaks CaSiO_3_ (84–655) and Ca_3_(Si_3_O_9_) (76–1846) of composite monoclinic deformations of wollastonite 2M were found. These compounds were introduced into the SCS sample together with the Carbofill E binder. At the same time, Cr (VI) was not detected in the form of Na_2_CrO_4_. It is likely that part of this salt in SCS samples initially reacts in an alkaline medium with Ca^2+^ ions present there, forming sparingly soluble CaCrO_4_ calcium chromate according to Equation (1):

Na_2_CrO_4_ + Ca(OH)_2_ → CaCrO_4_ +2KOH (1)

Furthermore, redox reactions take place in which sparingly soluble and less oxidized chromium compounds are formed. The part of hexavalent Cr, with an oxidation state of +6 in an alkaline environment, was reduced to hexagonal calcium chromium oxide Ca_5_(CrO4)_3_O_0.5_ (38–294), determined in the XRD study, with an oxidation state of +5 according to the equation:

(2) $$6{\mathrm{CaCrO}}_{4}+{\mathrm{CaO}}_{4}+3{\mathrm{Ca}}^{2+}+6\bar{e}\to 2{\mathrm{Ca}}_{5}{\left({\mathrm{CrO}}_{4}\right)}_{3}O_{0.5}.$$

The other part of Cr (VI) was reduced to the trivalent form of chromium with oxidation state of +3, practically insoluble and chemically inert, *rhombohedral* Cr_2_O_3_ chromium oxide (84–315) according to the equation:

(3) $$2{\mathrm{CaCrO}}_{4}+4H_{2}O+6\bar{e}\to {\mathrm{Cr}}_{2}O_{3}+\mathrm{CaO}+\mathrm{Ca}{\left(\mathrm{OH}\right)}_{2}+6{\mathrm{OH}}^{-}$$

XRD studies show that the immobilization of Cr (VI) in SCS was provided by the reaction of a well-soluble salt Na_2_CrO_4_ dissolution in water is 84.5 g/100 g at 25 °C) in an alkaline medium with Carbofill E components to form sparingly soluble and chemically inert chromium compounds that are difficult to wash out of soil samples.

## 4. Conclusions

The self-compacting soils (SCS) were made from sandy and clay soils randomly excavated from a local construction sites using Carbofill E as a binder. The SCS density, compressive strength, and water permeability properties produced show that modified soils are a suitable material to achieve the desired properties as designed. When sandy soil is used, the compressive strength of SCS is higher than that of clay soil, but it is necessary to use a higher amount of binder in sandy soil to achieve lower water permeability.

This study investigated the possibility of immobilizing hexavalent chromium in self-compacting soils (SCS) using Carbofill E as a binder. Samples of sandy and clay SCS with 10, 15, and 20% Carbofill E additives were used in the study; 500 to 100,000 mg of Cr (VI) in the form of Na_2_CrO_4_ were added to 1 kg of the SCS sample. Tests showed that the washing of chromium from undisturbed soil samples is more than a hundred times lower than that from crushed soil. At the same time, an increase in the amount of Carbofill E additive in SCS samples reduces the washing out of chromium from them. Therefore, when sandy SCS samples with the addition of 10% → 15% → 20% Carbofill E were washed, chromium was washed out of them as follows: 5.87 × 10^−2^ → 0.65 × 10^−2^ → 0.35 × 10^−2^ mg/kg from uncrushed samples and 18 → 1.1 → 0.591 mg/kg. The same chromium washing out relationship was observed from clay SCS samples. According to XRD data, the crystalline phases of *hexagonal* calcium chromium oxide Ca_5_(CrO_4_)_3_O_0.5_ (38–294) and *rhombohedral* chromium oxide Cr_2_O_3_ (84-315) were established. Therefore, it is likely that most of the chromium in the water-soluble form (Na_2_CrO_4_) is converted to practically insoluble Ca_5_(CrO_4_)_3_O_0.5_ and Cr_2_O_3_ chromium compounds during the formation of SCS samples, which contributes greatly to the efficiency of immobilization. Tests showed that SCSs, regardless of their nature, are suitable for immobilization, since after the samples are washed, more than 99% of the incorporated chromium remains in them. The use of SCS in construction offers the potential to have a significant economic and environmental impact, as well as the potential to immobilize hazardous waste in recycled soil. The effectiveness of the immobilization of individual soils contaminated with hazardous waste (such as petroleum products, hazardous polymeric substances, etc.) should be investigated in future research.

## Figures and Tables

**Table 1:** Sandy soil particle size distribution.

**Sieve Size, mm**	0.63	0.125	0.25	0.5	1	2	4	5.6
**Percentage** **Passing, %**	1.0	8.0	70.4	94.0	96.2	97.3	98.5	100

**Table 2:** Characterization of clay soil according to Atterberg’s limits.

Sample No.	Sample Moisture Content, %	Average Moisture Content, %	Plasticity Index (PI), %
Plastic limit (PL), %			25.36
1	25.93	25.48	
2	24.76		
Liquid limit (LL), %			
3	49.87	50.84	
4	51.26		

**Table 3:** Components used in the preparation of SCS mixtures and physical properties of the mixtures.

Soil Component	Carbofill E, %	Soil, %	Water, %	Diameter of Slump Flow, mm	Density of the Mixture, kg/m^3^
Sandy soil (Sa)	10	65	25	610	2000
	15	60	25	640	2036
	20	55	25	650	2077
Clay soil (Cl)	10	45	45	640	1631
	15	42	43	630	1644
	20	37	43	620	1699

**Table 4:** Dependence of the efficiency of immobilization of Cr (VI) as a percentage of the SCS type and the amount of Carbofill E.

Type of SCS	SCS Samples	Carbofill E, %		
		10	15	20
sandy	undisturbed	99.9980	99.9978	99.9988
	crushed	99.4	99.633	99.980
clay	undisturbed	99.9970	99.9983	99.99197
	crushed	97.847	99.655	99.806

3000 mg/kg Cr (VI) was added to the SCS samples.

## Data Availability

The data used to support the findings of this study can be made available from the corresponding author upon request.

## Supplementary Materials

The following supporting information can be downloaded at: https://www.mdpi.com/article/10.3390/ma15062335/s1, Table S1: The phase composition of sandy SCS samples with 10% Carbofill E and Cr (VI) 3000 mg/kg; Table S2: The phase composition of sandy SCS samples with 20% Carbofill E and Cr (VI) 3000 mg/kg; Table S3: The phase composition of sandy SCS samples with 10% Carbofill E and Cr (VI) 30,000 mg/kg; Table S4: The phase composition of clay SCS samples with 10% Carbofill E and Cr (VI) 3000 mg/kg; Table S5: The phase composition of clay SCS samples with 20% Carbofill E and Cr (VI) 3000 mg/kg.

## References

[1] Antoci A., Galeotti M., Sordi S. (2018). Environmental Pollution as Engine of Industrialization. Commun. Nonlinear Sci. Numer. Simul..

[2] Li X. (2019). Technical Solutions for the Safe Utilization of Heavy Metal-Contaminated Farmland in China: A Critical Review. Land Degrad. Dev..

[3] Rai P.K., Lee S.S., Zhang M., Tsang Y.F., Kim K.H. (2019). Heavy Metals in Food Crops: Health Risks, Fate, Mechanisms, and Management. Environ. Int..

[4] Rinklebe J., Antoniadis V., Shaheen S.M., Rosche O., Altermann M. (2019). Health Risk Assessment of Potentially Toxic Elements in Soils along the Central Elbe River, Germany. Environ. Int..

[5] Mao C., Song Y., Chen L., Ji J., Li J., Yuan X., Yang Z., Ayoko G.A., Frost R.L., Theiss F. (2019). Human Health Risks of Heavy Metals in Paddy Rice Based on Transfer Characteristics of Heavy Metals from Soil to Rice. CATENA.

[6] Korkmaz C., Ay Ö., Ersoysal Y., Köroğlu M.A., Erdem C. (2019). Heavy Metal Levels in Muscle Tissues of Some Fish Species Caught from North-East Mediterranean: Evaluation of Their Effects on Human Health. J. Food Compos. Anal..

[7] Shen X., Min X., Zhang S., Song C., Xiong K. (2020). Effect of Heavy Metal Contamination in the Environment on Antioxidant Function in Wumeng Semi-Fine Wool Sheep in Southwest China. Biol. Trace Elem. Res..

[8] Diaconu M., Pavel L.V., Hlihor R.M., Rosca M., Fertu D.I., Lenz M., Corvini P.X., Gavrilescu M. (2020). Characterization of Heavy Metal Toxicity in Some Plants and Microorganisms—A Preliminary Approach for Environmental Bioremediation. New Biotechnol..

[9] Adachi K., Kitada A., Fukami K., Murase K. (2020). Crystalline Chromium Electroplating with High Current Efficiency Using Chloride Hydrate Melt-Based Trivalent Chromium Baths. Electrochim. Acta.

[10] Inam A., Raza M.A., Hafeez M.A., Shah S.B., Ishtiaq M., Hassan M.H., Irfan M., Nasik A., Siddique I., Butt O.M. (2020). Effect of Voltage and Spray–off Distance of Electric-Arc Spray Technique on Surface Properties of Nickel–Chrome (Ni–Cr) Coating Developed on 304L Stainless Steel. Mater. Res. Express.

[11] Mekicha M.A., de Rooij M.B., Matthews D.T.A., Pelletier C., Jacobs L., Schipper D.J. (2020). The Effect of Hard Chrome Plating on Iron Fines Formation. Tribol. Int..

[12] Ageev E.V., Altukhov A.Y., Korolyov M.S. (2020). The Phase Composition of Products from Electro-Erosive Cobalto-Chrome Powders, Obtained by Additive Technologies. Solid State Phenom..

[13] Ding W., Pang X., Ding Z., Tsang D.C.W., Jiang Z., Shi B. (2020). Constructing a Robust Chrome-Free Leather Tanned by Biomass-Derived Polyaldehyde via Crosslinking with Chitosan Derivatives. J. Hazard. Mater..

[14] Jiang Y., Li J., Liu F., Zhang Z., Li Z., Yang M., Li L. (2019). The Effects of Surface Modification Using O2 Low Temperature Plasma on Chrome Tanning Properties of Natural Leather. J. Ind. Text..

[15] Kabir S.M.F., Chakraborty S., Hoque S.M.A., Mathur K. (2019). Sustainability Assessment of Cotton-Based Textile Wet Processing. Clean Technol..

[16] Langård S., Costa M. (2015). Chromium. Handbook on the Toxicology of Metals: Fourth Edition.

[17] Khodavirdipour A., Haddadi F., Keshavarzi S. (2020). Chromium Supplementation; Negotiation with Diabetes Mellitus, Hyperlipidemia and Depression. J. Diabetes Metab. Disord..

[18] Behrouz V., Dastkhosh A., Sohrab G. (2020). Overview of Dietary Supplements on Patients with Type 2 Diabetes. Diabetes Metab. Syndr..

[19] Abd-El A., Mohammed R., Abd W., Abd-Elwahab E. (2020). The Potential Protective Role of Vitamin E and Selenium against Sub-Chronic Toxicity of Hexavalent Chromium on the Testis of Adult Male Albino Rats. Ain Shams J. Forensic Med. Clin. Toxicol..

[20] Chen Q.Y., Murphy A., Sun H., Costa M. (2019). Molecular and Epigenetic Mechanisms of Cr(VI)-Induced Carcinogenesis. Toxicol. Appl. Pharmacol..

[21] Yan K., Liu Z., Li Z., Yue R., Guo F., Xu Z. (2019). Selective Separation of Chromium from Sulphuric Acid Leaching Solutions of Mixed Electroplating Sludge Using Phosphate Precipitation. Hydrometallurgy.

[22] Reyes-Serrano A., López-Alejo J.E., Hernández-Cortázar M.A., Elizalde I. (2020). Removing Contaminants from Tannery Wastewater by Chemical Precipitation Using CaO and Ca(OH)2. Chinese J. Chem. Eng..

[23] Jang E.H., Pack S.P., Kim I., Chung S. (2020). A Systematic Study of Hexavalent Chromium Adsorption and Removal from Aqueous Environments Using Chemically Functionalized Amorphous and Mesoporous Silica Nanoparticles. Sci. Rep..

[24] Pakade V.E., Tavengwa N.T., Madikizela L.M. (2019). Recent Advances in Hexavalent Chromium Removal from Aqueous Solutions by Adsorptive Methods. RSC Adv..

[25] Thomas T.N., Ahammed M.M. (2020). Removal of Chromium Using Water Treatment Sludge. Lect. Notes Civ. Eng..

[26] Kahraman H.T., Pehlivan E. (2019). Evaluation of Anion-Exchange Resins on the Removal of Cr(VI) Polluted Water: Batch Ion-Exchange Modeling. Arab. J. Geosci..

[27] Ye Z., Yin X., Chen L., He X., Lin Z., Liu C., Ning S., Wang X., Wei Y. (2019). An Integrated Process for Removal and Recovery of Cr(VI) from Electroplating Wastewater by Ion Exchange and Reduction–Precipitation Based on a Silica-Supported Pyridine Resin. J. Clean. Prod..

[28] Noah N.F.M., Sulaiman R.N.R., Othman N., Jusoh N., Rosly M.B. (2020). Extractive Continuous Extractor for Chromium Recovery: Chromium (VI) Reduction to Chromium (III) in Sustainable Emulsion Liquid Membrane Process. J. Clean. Prod..

[29] Wei X.Z., Gan Z.Q., Shen Y.J., Qiu Z.L., Fang L.F., Zhu B.K. (2019). Negatively-Charged Nanofiltration Membrane and Its Hexavalent Chromium Removal Performance. J. Colloid Interface Sci..

[30] Chen J., Wang Y., Wang H., Zhou S., Wu H., Lei X. (2016). Detoxification/Immobilization of Hexavalent Chromium Using Metakaolin-Based Geopolymer Coupled with Ferrous Chloride. J. Environ. Chem. Eng..

[31] Bae S., Hikaru F., Kanematsu M., Yoshizawa C., Noguchi T., Yu Y., Ha J. (2018). Removal of Hexavalent Chromium in Portland Cement Using Ground Granulated Blast-Furnace Slag Powder. Materials.

[32] Ferri M., Campisi S., Scavini M., Evangelisti C., Carniti P., Gervasini A. (2019). In-Depth Study of the Mechanism of Heavy Metal Trapping on the Surface of Hydroxyapatite. Appl. Surf. Sci..

[33] Ding X., Shan Z., Long Z., Chen Z. (2020). Utilization of Collagen Protein Extracted from Chrome Leather Scraps as a Set Retarders in Gypsum. Constr. Build. Mater..

[34] Cristelo N., Coelho J., Oliveira M., Consoli N.C., Palomo Á., Fernández-Jiménez A. (2020). Recycling and Application of Mine Tailings in Alkali-Activated Cements and Mortars—Strength Development and Environmental Assessment. Appl. Sci..

[35] Liao C.Z., Tang Y., Lee P.H., Liu C., Shih K., Li F. (2017). Detoxification and Immobilization of Chromite Ore Processing Residue in Spinel-Based Glass-Ceramic. J. Hazard. Mater..

[36] He L., Li B., Lin Z., Ning P., Shen Z. (2019). Mechanism of Dry Detoxification of Chromium Slag by Carbon Monoxide. Environmental Chemistry Letters.

[37] Zhou M., Liu X., Chen X., Gao P. (2021). Study on Strength, Water Stability, Shrinkage, and Microstructure of CFB Slag Modified Cement Stabilized Clay. Materials.

[38] Zabielska-Adamska K., Wasil M., Dobrzycki P. (2021). Resilient Response of Cement-Treated Coarse Post-Glacial Soil to Cyclic Load. Materials.

[39] Ouellet-Plamondon C.M., Habert G. (2016). Self-Compacted Clay Based Concrete (SCCC): Proof-of-Concept. J. Clean. Prod..

[40] Agilent Technologies Flame Atomic Absorption Spectrometry Analytical Methods. www.agilent.com.

[41] Ministry of Health of the Republic of Lithuania V-114 Dėl Lietuvos Higienos Normos HN 60:2004 “Pavojingų Cheminių Medžiagų Didžiausios Leidžiamos Koncentracijos Dirvožemyje” [Lithuanian Hygiene Standard HN 60: 2015 “Limit Values for Hazardous Chemicals in Soil”]. https://e-seimas.lrs.lt/portal/legalAct/lt/TAD/TAIS.228693/EffUOaxQaa?jfwid=-9faifhqeq.

[42] Tantawy M.A., El-Roudi A.M., Salem A.A. (2012). Immobilization of Cr(VI) in Bagasse Ash Blended Cement Pastes. Constr. Build. Mater..

