# Supplementary material for: Immobilization of Hexavalent Chromium Using Self-Compacting Soil Technology
Source: Materials (Basel). 2022 Mar 21;15(6):2335. doi: 10.3390/ma15062335 (PMC8953853; doi:10.3390/ma15062335)
Supplement: Supplementary file 1 [file materials-15-02335-s001.zip › materials-1595892-supplementary.pdf]

Article

Immobilization of Hexavalent Chromium Using Self-Compacting Soil Technology

Zymantas Rudzionis [1,*], Arunas Aleksandras Navickas [1], Gediminas Stelmokaitis [1] and Remigijus Ivanauskas [2]

[1] Faculty of Civil Engineering and Architecture, Kaunas University of Technology, Studentų str. 48, 51367 Kaunas, Lithuania; arunas.navickas@ktu.lt (A.A.N.); gediminas.stelmokaitis@ktu.lt (G.S.)

[2] Faculty of Chemical Technology, Kaunas University of Technology, Radvilėnų pl. 19, 50254 Kaunas, Lithuania; remigijus.ivanauskas@ktu.lt

* Correspondence: zymantas.rudzionis@ktu.lt

Abstract: A study of immobilization of hexavalent chromium in the form of Na_2CrO_4 salt by self-compacting soils (SCS) is presented. Carbofill E additive was used as SCS binder. The efficiency of immobilization of Cr (VI) was evaluated by washing out chromium compounds from SCS samples. The influence of the nature of the soil and the content of Carbofill E and Na_2CrO_4 in the SCS samples on the efficiency of Cr (VI) immobilization was studied. It was found that the nature of the soil and the content of Carbofill E in the SCS samples affect the immobilization of Cr (VI). Moreover, increasing the Carbofill E content in SCS samples further increases Cr (VI) immobilization. X-ray diffraction studies of the samples with immobilized hexavalent chromium showed that part of the sample transforms from a readily soluble form of salt into oxide forms of chromium and calcium-chromium, which are practically insoluble in water.

Keywords: immobilization; hexavalent chromium; self-compacting soil

Table S1. The phase composition of sandy self-compacting soils (SCS) samples with 10% Carbofill E and Cr (VI) 3000 mg/kg.

Compound	2θ (°)	*d*-spacing (Å)	
		Observed values	JCPDS data
Quartz (*hexagonal*) SiO_2 83-539 (○)	20.83	4.26	4.26
	26.59	3.35	3.35
	36.52	2.46	2.46
	39.43	2.28	2.28
	40.26	2.24	2.24
	42.42	2.13	2.13
	45.75	1.98	1.98
	50.11	1.82	1.82
	54.81	1.67	1.67
	59.96	1.54	1.54
	67.71	1.38	1.38
Wollastonite 2M (*monoclinic*) $CaSiO_3$ 84-655 (▲)	17.97	4.93	4.92
	27.39	3.25	3.25
Wollastonite 2M (*monoclinic*) $Ca_3(Si_3O_9)$ 76-1846 (△)	25.62	3.47	3.47
	27.94	3.19	3.19
	29.36	3.04	3.04
Calcium Chromium Oxide (*hexagonal*) $Ca_5(CrO_4)_3O_{0.5}$ 38-294 (●)	30.90	2.89	2.89
	68.31	1.37	1.37

Table S2. The phase composition of sandy SCS samples with 20% Carbofill E and Cr (VI) 3000 mg/kg.

Compound	2θ (°)	*d*-spacing (Å)	
		Observed values	JCPDS data
Quartz (*hexagonal*) SiO_2 83-539 (○)	20.83	4.26	4.26
	26.59	3.35	3.35
	36.52	2.46	2.46
	39.43	2.28	2.28
	40.26	2.24	2.24
	42.42	2.13	2.13
	50.11	1.82	1.82
	54.81	1.67	1.67
	59.96	1.54	1.54
	67.71	1.38	1.38
Wollastonite 2M (*monoclinic*) $CaSiO_3$ 84-655 (▲)	17.97	4.93	4.92
	50.77	1.80	1.80
	55.34	1.66	1.66
Wollastonite 2M (*monoclinic*) $Ca_3(Si_3O_9)$ 76-1846 (△)	29.36	3.04	3.04
Calcium Chromium Oxide (*hexagonal*) $Ca_5(CrO_4)_3O_{0.5}$ 38-294 (●)	30.90	2.89	2.89
	68.31	1.37	1.37
Chromium Oxide (*rhombohedral*) Cr_2O_3 84-315 (✱)	33.91	2.64	2.64

Table S3. The phase composition of sandy SCS samples with 10% Carbofill E and Cr (VI) 30,000 mg/kg.

Compound	2*θ* (°)	*d*-spacing (Å)	
		Observed values	JCPDS data
Quartz (*hexagonal*) SiO_2 83-539 (○)	20.83	4.26	4.26
	26.59	3.35	3.35
	36.52	2.46	2.46
	39.43	2.28	2.28
	40.26	2.24	2.24
	42.42	2.13	2.13
	45.75	1.98	1.98
	50.11	1.82	1.82
	59.96	1.54	1.54
	67.71	1.38	1.38
Wollastonite 2M (*monoclinic*) $CaSiO_3$ 84-655 (▲)	17.97	4.93	4.92
	41.13	2.19	2.19
	55.34	1.66	1.66
Wollastonite 2M (*monoclinic*) $Ca_3(Si_3O_9)$ 76-1846 (△)	25.62	3.47	3.47
	29.36	3.04	3.04
Calcium Chromium Oxide (*hexagonal*) $Ca_5(CrO_4)_3O_{0.5}$ 38-294 (●)	30.90	2.89	2.89
	68.31	1.37	1.37
Chromium Oxide (*rhombohedral*) Cr_2O_3 84-315 (✱)	33.91	2.64	2.64

Table S4. The phase composition of clayey SCS samples with 10% Carbofill E and Cr (VI) 3000 mg/kg.

Compound	2*θ* (°)	*d*-spacing (Å)	
		Observed values	JCPDS data
Quartz (*hexagonal*) SiO_2 83-539 (○)	20.82	4.26	4.26
	26.59	3.35	3.35
	36.52	2.46	2.46
	39.43	2.28	2.28
	40.26	2.24	2.24
	42.42	2.13	2.13
	45.75	1.98	1.98
	50.11	1.82	1.82
	54.81	1.67	1.67
	59.96	1.54	1.54
	67.71	1.38	1.38
Wollastonite 2M (*monoclinic*) $CaSiO_3$ 84-655 (▲)	27.39	3.25	3.25
	47.48	1.91	1.91
	48.47	1.87	1.87
Wollastonite 2M (*monoclinic*) $Ca_3(Si_3O_9)$ 76-1846 (△)	29.36	3.04	3.04
Calcium Chromium Oxide (*hexagonal*) $Ca_5(CrO_4)_3O_{0.5}$ 38-294 (●)	30.90	2.89	2.89
	43.11	2.10	2.10
	68.31	1.37	1.37

Table S5. The phase composition of clayey SCS samples with 20% Carbofill E and Cr (VI) 3000 mg/kg.

Compound	2θ (º)	*d*-spacing (Å)	
		Observed values	JCPDS data
Quartz (*hexagonal*) SiO_2 83-539 (○)	20.82	4.26	4.26
	26.59	3.35	3.35
	36.52	2.46	2.46
	39.43	2.28	2.28
	40.26	2.24	2.24
	42.42	2.13	2.13
	45.75	1.98	1.98
	50.11	1.82	1.82
	59.96	1.54	1.54
	67.71	1.38	1.38
Wollastonite 2M (*monoclinic*) $CaSiO_3$ 84-655 (▲)	17.97	4.93	4.92
	47.48	1.91	1.91
	48.47	1.87	1.87
Wollastonite 2M (*monoclinic*) $Ca_3(Si_3O_9)$ 76-1846 (△)	29.36	3.04	3.04
Calcium Chromium Oxide (*hexagonal*) $Ca_5(CrO_4)_3O_{0.5}$ 38-294 (●)	30.90	2.89	2.89
	43.11	2.10	2.10
	68.31	1.37	1.37
